# Rev. David Nelson, M.D., D.D.

**Published:** 1861-07

**Authors:** 


###  HALL'S JOURNAL OF HEALTH.

Our Legitimate Scope is almost boundless: for whatever begets pleasurable and harmless feelings, promotes Health; and whatever induces disagreeable sensations, engenders Disease.

WE AIM TO SHOW HOW DISEASE MAY BE AVOIDED, AND THAT IT IS BEST, WHEN SICKNESS COMES, TO TAKE NO MEDICINE WITHOUT CONSULTING A PHYSICIAN.

Vol. VIII.] JULY, 1861. [No. 7.

### REV. DAVID NELSON, M.D., D.D.,

### THE AUTHOR OF THE CAUSE AND CURE OF INFIDELITY.

NEXT to the blessing of an intelligent and pious mother, is the privilege of knowing, in early life, a truly great and good man. The spectacle of intellect and piety embodied thus before us, lingers as a consecrated thing in the memory of after years, and leaves an indelible impression on the character. It was my rare privilege, at an early period of life, to form, under peculiar circumstances, an intimate acquaintance with the late Dr. Nelson, as intimate as could well exist between a youth of twenty and a man in the full maturity of his powers, in the zenith of his fame, and amidst the incessant activities of a most laborious and successful ministry. With every opportunity of unconstrained association, seeing him, hearing him, in public and in private, weekly, daily, in the pulpit, at the family altar, in the domestic circle, observing his habits of thought, his method of sermonizing, the books he read, the opinions he held, the various peculiarities of his intellect and character, if there be presented, in these sketches, an erroneous conception of this man, it must be attributed to want of capacity to appreciate him justly, and not to want of advantages for thorough knowledge.

This acquaintance was renewed from time to time by person al intercourse, during a period of fifteen or eighteen years, and after seeing and hearing many of the most distinguished ministers and theologians at home and abroad, the impression remains distinct and vivid that he was not only the prince of preachers, but the noblest of men, and the former precisely because he was the latter. He was a model of apostolic simplicity, sincerity, earnestness, and I might add, of apostolic grandeur. The holiness and greatness of the man would have awed you, had there not been an inexpressible human naturalness about him—a gentle human sympathy, sometimes a child-like *naïvete*, which fascinated and reässured you. You felt that there was the bond of a common nature between you. He, too, was a man; and as he talked, whether in his gayer or more solemn moods, you felt that the very depths of his being were laid open before you, and you held converse with a genuine human soul. His countenance, in some of its nobler aspects, was often recalled, in after-life, by that of Luther, in the best portraits of the great reformer; with less of breadth and comprehensiveness, indeed, yet never, even in that large Teutonic heart, was there a richer fund of humor, or a keener, quicker relish for merriment and harmless fun. His appreciation of the loveliness of female piety, as in all lofty minds, was exquisite, and in the society of the young daughters of his friends, just passing into womanhood, in the exuberant gayety of opening life, that grave and thoughtful countenance would sometimes relax into playful merriment; he would recite whole cantoes of their favorite poems, discuss the heroes and heroines of romance, kindle into enthusiasm as he dilated on the character of Rowena or Die Vernon, and shake his sides with laughter over the Fat Friar of Copmanhurst.

He had a profound knowledge of MAN and MEN, a profound comprehension of the universal elements of our nature, and a quick insight into individual character. To those who knew him only at a distance as the lofty orator, in public, in private only as the rapt and silent and meditative thinker, this may appear a paradox. But the very vividness of his interest in spiritual things made him only more widely awake and keenly sensitive in regard to all that concerned man's eternal destiny. Quick to perceive, and acute to analyze each aspect of human nature that might harmonize with or oppose the great object of his ministry. Hence, it may be doubted whether he ever addressed, in his better days, an assembly of his fellow-men, without some deep and manifest impression, or ever personally approached a human being who did not feel and recognize his power. The man who touched and fascinated Marshall, and Breckinridge, and Denny, and Crittenden, and Robertson, and Ross, and Grundy, and John Bell, so different, each from the other, and all so different from himself, must have known and touched our nature at innumerable points. He would, in like manner, have touched Tholuck, and Neander, and Schleiermacher, and Steffins, and Chalmer, and Vinet. They would have smiled, perhaps, at his ignorance of some departments of human science. He would have pitied their comparative ignorance of all that it most behooves man to know. All would have recognized his immeasurable superiority in all the elements of moral greatness. The attributes that constitute true greatness, the thoughts and interests that stir man's soul to its profoundest depths, are not peculiar to any age or nation. He would have been recognized in London, or Berlin, if he had spoken German, as cordially and enthusiastically as in Baltimore, or Danville, or New-York.

The grandeur of the themes which were the habitual subject of his thoughts, gave a corresponding grandeur to his character; and the intensity with which he studied them communicated such habitual and healthy activity to his vigorous understanding, and such vivid and condensed power to his language, that he looked down, not with arrogance, but with pity, upon the trivial pursuits, and listened, with wonder at their weakness, to the ordinary efforts of even highly-gifted men. On his return from a visit to Washington City, he said, "I had known Mr. Grundy and John Bell in Tennessee. Grundy took me to the Senate Chamber, and introduced me to Clay, Webster, Calhoun, and others. I could not help pitying those men, that they wasted such talents and so much time about such trifles. I heard them all speak, even on the trifling subjects they discussed;" the speeches seemed to him about worthy of the subjects, and were heard with surprise and pain.

"James," said he, turning to a youth then present. and who may remember the conversation still, "James, I hope you will not spend your life in making marks on the sand, or scratching in the ashes like ——;" he called the name of the most distinguished of them all. So trivial, and even pitiful in his views, so unworthy of an immortal being, and so belittling to the intellect itself, were all the highest objects of earthly ambition, and all the efforts which those objects or that ambition could inspire. It was this habitual, unassumed, and unassuming elevation of character and purpose, united with the most unfeigned humility and simplicity of character, which gave the delicate point to the remark of one who loved and admired him above all living men when, turning abruptly to him, he said, in seeming censure: "Dr. Nelson, you are the most ambitious man I ever saw—I do believe, the most ambitious man *in the world*." "Why?" asked the Doctor, startled, and fearing, perhaps, that the keen eye of a faithful friend had detected the remains of a worldly pride, which he had hoped was long since subdued. "Because you are satisfied with nothing this world can give—nothing less will content you than a *kingdom, and a throne, and a crown all in heaven*." That kingdom, that throne, that crown of glory, were ever before his eyes, and beneath the power of that transcendent vision, all the energies of his understanding and all the feelings of his heart were aroused to their utmost activity, and concentrated on the one great object of his life. He lived, and moved, and had his being amidst the realities of the eternal world, and walked by faith amidst them, as if he saw them palpably before him in the broad light of day, and with the sober certainty of waking vision.

He went from the closet to the pulpit with the solemn impression of these great realities vivid and fresh upon him. His words of exhortation were like a voice from heaven; his tones of warning or denunciation like the trump of God. "*It is the blood earnestness of the man!*" said Dr. Mason, when asked: "What is the secret of Chalmers' power?"

We are told by some that knowledge is power, that genius is power, that energy of will is power. But the great lessons which Nelson has left behind for the instruction of his own and succeeding generations is, *that holiness is power*, that *faith is power*, that a life imbued with the spirit of the Gospel, and consecrated to its service, a soul glowing with its hopes, sanctified by its truths, sustained, impelled, exalted by its sacred influences, and in habitual communication with its Author—*this is power—the mightiest power on earth*—and by God's blessing, well nigh irresistible.

What wonder if that herculean form sank down, at last, beneath those stupendous labors; and that gigantic intellect shook down, at last, by the intensity of its own action, the frail tenement it inhabited, and if not overwhelmed, was, at least, partially obscured amidst its ruins. To say this is only to enroll him amongst the mighty army of martyrs—that hallowed band of consecrated and heroic spirits, whom the zeal of God's house had consumed. A calculating prudence might have prolonged that life, and preserved, for a season, those powers for the Church and the world. But that life, so delicately nursed, had not been Nelson's life; those energies, so cautiously restrained and securely fettered, had not been his. It seems the inevitable destiny of the world's best and greatest men, to be consumed in diffusing light and blessings to others. The eagle's pinion wings the shaft that quivers in the eagle's heart. Only for the martyr spirit are reserved the martyr's crown of glory above, and the martyr's deathless memory on earth. I witnessed the first indication, at any rate, the first public and decisive indications of that malady, which afterwards obscured the brilliancy of his genius, without ever destroying its equipoise, however, or disturbing the serenity of his Christian hope. A Senator from Mississippi was on a visit to Danville, his native place. He had just entered upon his political career at Washington, when the premonitory symptoms appeared of that hereditary consumption which consigned him to his grave. He was outwardly, a cold, phlegmatic man, though with strong, deep feelings, reserved, some would say haughty; imbued with the skeptical sentiments which, nurtured by the writings of Hume and Gibbon, and the high authority of Mr. Jefferson, had pervaded our most intelligent society.

With that quickness of propriety and ready perception of character which marked all his intercourse with men, Nelson never sought the society of this gentleman, nor intruded on his privacy. He agreed, however, to preach at the house of a common friend, where the visitor was expected to be present, and the discourse was designed for his especial benefit. In the midst of one of those impassioned bursts of eloquence which all of us remember and none can describe, in which fact and argument, reason and imagination, sublimity and pathos, blended in inexplicable combination, at the point where genius trembles almost on the verge of inspiration, he paused, drew his hand slowly across his brow, and calmly said, with an expression rather of surprise than alarm: "A *strange* oblivion has passed over me." The great mind had sunk beneath the vastness of its own conception; amidst the full swell of that majestic melody, the cords from which it issued burst from the intensity of their own vibrations. Man can not rival the angels, though he may "have moments *like* their highest." There was no thought, in any mind, of what we now call a failure; no expression, on any countenance, of mortification, scarcely of sadness, but of wonder, rather perhaps allied to *awe—awe* as of some portentous mystery—the great continuity of nature broken off, and a black chasm before you, Men did not sympathize with Nelson. They revered him as an old apostle, or one of the prophets as, apart from inspiration, one might bow reverently before Paul, or tenderly love St. John. At least, so it seemed to one who loved and revered him.—MATHETES—*Center College Magazine.*

